# Is the outcome of elective vs non-elective patients undergoing transcatheter aortic valve implantation different? Results of a single-centre, observational assessment of outcomes at a large university clinic

**DOI:** 10.1186/s12872-023-03317-5

**Published:** 2023-06-10

**Authors:** Steffen Wundram, Hatim Seoudy, Johannes C. Dümmler, Lukas Ritter, Johanne Frank, Thomas Puehler, Georg Lutter, Matthias Lutz, Mohammed Saad, Peter Bramlage, Janarthanan Sathananthan, David A. Wood, Sandra B. Lauck, Norbert Frey, Derk Frank

**Affiliations:** 1 Department of Internal Medicine III, Cardiology, Angiology and Critical Care, University Hospital Schleswig-Holstein, Arnold-Heller-Str.3, Haus K3, 24105 Kiel, Germany; 2 DZHK (German Centre for Cardiovascular Research), Partner Site Hamburg/Kiel/Lübeck, Kiel, Germany; 3 Department of Anaesthesiology and Intensive Care Medicine, University Hospital Schleswig-Holstein, Campus Kiel, Kiel, Germany; 4 Department of Cardiac and Vascular Surgery, University Hospital Schleswig-Holstein, Campus Kiel, Kiel, Germany; 5 Institute for Pharmacology and Preventive Medicine, Bahnhofstrasse 20, 49661 Cloppenburg, Germany; 6 Centre for Cardiovascular Innovation – Centre d’Innovation Cardiovasculaire, St Paul’s and Vancouver General Hospitals, University of British Columbia, Vancouver, Canada; 7 School of Nursing, University of British Columbia, Vancouver, Canada; 8 University Hospital of Heidelberg, Cardiology, , Heidelberg, Germany; 9 DZHK (German Centre for Cardiovascular Research), Partner Site Heidelberg/Mannheim, Heidelberg, Germany

**Keywords:** Transcatheter aortic valve implantation, Aortic stenosis, Fast-track, Coordinator, Patient care

## Abstract

**Background:**

Transcatheter aortic valve implantation (TAVI) can either be conducted as an elective (scheduled in advance) or a non-elective procedure performed during an unplanned hospital admission. The objective of this study was to compare the outcomes of elective and non-elective TAVI patients.

**Methods:**

This single-centre study included 512 patients undergoing transfemoral TAVI between October 2018 and December 2020; 378 (73.8%) were admitted for elective TAVI, 134 (26.2%) underwent a non-elective procedure. Our TAVI programme entails an optimized fast-track concept aimed at minimizing the total length of stay to ≤ 5 days for elective patients which in the German healthcare system is currently defined as the minimal time period to safely perform TAVI. Clinical characteristics and survival rates at 30 days and 1 year were analysed.

**Results:**

Patients who underwent non-elective TAVI had a significantly higher comorbidity burden. Median duration from admission to discharge was 6 days (elective group 6 days versus non-elective group 15 days; *p* < 0.001), including a median postprocedural stay of 5 days (elective 4 days versus non-elective 7 days; *p* < 0.001). All-cause mortality at 30 days was 1.1% for the elective group and 3.7% for non-elective patients (*p* = 0.030). At 1 year, all-cause mortality among elective TAVI patients was disproportionately lower than in non-elective patients (5.0% versus 18.7%, *p* < 0.001). In the elective group, 54.5% of patients could not be discharged early due to comorbidities or procedural complications. Factors associated with a failure of achieving a total length of stay of ≤ 5 days comprised frailty syndrome, renal impairment as well as new permanent pacemaker implantation, new bundle branch block or atrial fibrillation, life-threatening bleeding, and the use of self-expanding valves. After multivariate adjustment, new permanent pacemaker implantation (odds ratio 6.44; 95% CI 2.59–16.00), life-threatening bleeding (odds ratio 4.19; 95% confidence interval 1.82–9.66) and frailty syndrome (odds ratio 5.15; 95% confidence interval 2.40–11.09; all *p* < 0.001, respectively) were confirmed as significant factors.

**Conclusions:**

While non-elective patients had acceptable periprocedural outcomes, mortality rates at 1 year were significantly higher compared to elective patients. Approximately only half of elective patients could be discharged early. Improvements in periprocedural care, follow-up strategies and optimized treatment of both elective and non-elective TAVI patients are needed.

## Background

Severe symptomatic aortic stenosis (AS) is a life-threatening disease and surgical aortic valve replacement has been the mainstay of therapy for the last decades. Transcatheter aortic valve implantation (TAVI) has been developed as a treatment alternative using vascular access. While TAVI was initially restricted to patients at high or prohibitive surgical risk, it has demonstrated remarkable improvements of clinical outcomes in AS patients across the whole spectrum of surgical risk and has evolved as a viable treatment alternative for the majority of patients [1]. As there is a constant increase in TAVI procedures, the development and implementation of efficient TAVI programmes has become both a challenge and a priority [2–4].

TAVI can either be conducted as a pre-planned elective or as a non-elective (urgent) procedure during an unplanned hospital admission. Previous studies have reported conflicting results with regard to periprocedural complication rates as well as 30-day and 1-year outcomes of elective and non-elective patients [5, 6]. However, data on outcomes of elective and non-elective patients in the context of a contemporary TAVI programme are still limited. Efficient programmes require the adoption of processes that facilitate appropriate patient selection and evaluation, procedural success, and early discharge from hospital, without compromising clinical outcomes [2–4, 7]. At our institution, a fast-track protocol was implemented with the goal of providing a streamlined TAVI pathway to facilitate rapid mobilisation and early discharge. In particular, our TAVI programme aims at minimizing the total length of stay to ≤ 5 days for elective patients which in the German healthcare system is currently viewed as the minimal time period required to safely perform TAVI [8]. It entails optimisation at all levels, including pre-, peri- and postprocedural care [9]. The objective of the present study was to report (1) the clinical features and outcomes of elective versus non-elective TAVI patients at our centre and (2) to identify factors associated with a failure of early discharge in elective TAVI patients.

## Methods

### Study design

A total of 512 consecutive patients who underwent transfemoral TAVI at our centre between October 2018 and December 2020 were prospectively enrolled in an observational TAVI database. The decision to perform TAVI was based on the evaluation by our heart team following a comprehensive diagnostic workup.

All patients who underwent pre-planned TAVI were defined as elective patients, irrespective of their comorbidities. In elective patients, administrative procedures and standard preprocedural workup were conducted on the day of admission. After prior discussion by our heart team, a small subset of patients received a high-resolution multidetector computed tomography scan or a planned percutaneous coronary intervention on the day of admission due to logistic reasons (i.e. patients who were required to travel long distances to be admitted to our center). In patients who underwent computed tomography on the day of admission, the procedural plan (transfemoral versus non-transfemoral access) was determined on the same day (i.e. one day prior to the TAVI procedure). TAVI was performed within 48 h after admission. All elective patients were treated according to our fast-track protocol in order to limit the total length of stay to ≤ 5 days. In contrast, patients who underwent TAVI during an unplanned hospital admission and who after assessment of the heart team were considered unable to be treated electively, were defined as non-elective.

### Elective TAVI with a fast-track protocol

At our centre, elective TAVI patients are enrolled with a fast-track protocol which includes appropriate care with a streamlined approach while ensuring safe TAVI that allows for rapid mobilisation, timely discharge and minimal out-of-hospital risk. A central element of our program is a dedicated TAVI coordinator. The TAVI coordinator provides continuity of care, facilitates and coordinates optimized patient assessment and triage, communicates with patients and members of the heart team, and manages the waitlist. Moreover, the TAVI coordinator is responsible for planning diagnostic tests, gathering results for consideration by the heart team, scheduling TAVI procedures, and organizing patient discharge with an appropriate level of care. The position of a TAVI coordinator is increasingly implemented at TAVI centers in Germany [10]. In addition to a comprehensive preprocedural assessment of the patient using anatomical, functional and diagnostic parameters, the fast-track protocol at our centre incorporates various peri- and postprocedural components. The procedural approach includes (1) the use of local anaesthesia as a default strategy (i.e. no conscious sedation), (2) minimal skin preparation, (3) an open visual field between the patient and the implanter and anaesthesiologist, (4) ultrasound guided vascular access, (5) minimal use of invasive lines, (6) removal of all arterial sheaths in the hybrid operating room, (7) target activated clotting time of 150–200 s at the end of the procedure and (8) an immediate transthoracic echocardiogram to identify potential complications such as pericardial effusion/tamponade after TAVI. Whenever possible the temporary pacing wire is removed in the hybrid operating room. Closure of the vascular access is usually performed using two Perclose ProGlide™ vascular closure systems (Abbott Laboratories, Chicago, IL, USA). After the procedure, patients are monitored in a specialised Intermediate Care Unit for a minimum of 6 h. In the absence of new conduction disturbances or vascular access site complications, patients are mobilised within 6–8 h after the procedure and transferred to a regular ward, where they remain under telemetry monitoring for approximately 72 h.

### Procedural details

All TAVI procedures were performed using either SAPIEN 3 and SAPIEN 3 Ultra transcatheter heart valves (Edwards Lifesciences, Irvine, California) or CoreValve Evolut R/PRO devices (Medtronic, Minneapolis, Minnesota). Optimal type and size of transcatheter heart valve were determined using preprocedural computed tomography measurements evaluated using the 3mensio Structural Heart software (3mensio Medical Imaging BV, Bilthoven, The Netherlands). Pre-dilatation and post-dilatation were left to the physician’s discretion. During TAVI, unfractionated heparin was administered to achieve an activated clotting time of 250–300 s.

### Data collection

Written informed consent was obtained from each patient. The study was approved by the Ethics Committee at the University of Kiel (protocol code D 529/16) and the investigation conforms with the principles outlined in the Declaration of Helsinki [11]. Patient data and blood samples were collected 1–3 days prior to TAVI. Follow-up after discharge usually included a visit to our cardiology outpatient clinic 1–3 months after TAVI, as well as an annual telephone call follow-up. Here we report outcomes of up to 1 year.

### Patient and public involvement

It was not appropriate or possible to involve patients or the public in the design, or conduct, or reporting, or dissemination plans of our research.

### Statistical analyses

Continuous data were assessed for normality using the Shapiro–Wilk test and did not show a normal distribution. Accordingly, all continuous data were presented as median and interquartile range (IQR). Categorical data were summarised as frequencies (percentage, %). Data were analysed using the Mann–Whitney-U and Student’s test, as applicable, as well as the χ^2^-test. Fisher’s exact test was used when there were few observations (frequency less than 10 for an individual cell). Outcomes were presented based on the VARC-3 system [8]. Adjusted odds ratios (ORs) were calculated using a backward selection, multivariable logistic regression model in order to assess factors associated with an inability to follow a fast-track approach and presented together with 95% confidence interval (CIs). Rates of the composite endpoint of cardiovascular death, non-fatal stroke and non-fatal myocardial infarction were assessed using Kaplan–Meier analyses and the log-rank test. Factors associated with a failure of the fast-track TAVI protocol were expressed as ORs. All tests were two-sided. Probability values less than or equal to 0.05 were considered significant. All statistical analyses were performed using R software, Version 4.0.4, and GraphPad PRISM, version 8.

## Results

A total of 512 patients who underwent transfemoral TAVI at our centre between October 2018 and December 2020 were prospectively enrolled in an observational TAVI database, of whom 378 (73.8%) were admitted for an elective procedure, while 134 patients (26.2%) underwent non-elective TAVI. The reasons for non-elective TAVI included acute decompensation of AS (85.1%) and/or high symptom burden (32.8%) as well as acute coronary syndrome (22.4%) preventing an elective procedure after assessment of our heart team.

### Patient characteristics

The median age of the study population was 82 years and 46.3% were female (Table 1) with a higher proportion of female patients in the non-elective group (53.7% versus 43.7%; *p* = 0.044). Non-elective patients had a greater comorbidity burden, with higher rates of atrial fibrillation (AF), peripheral artery disease (PAD) and renal impairment compared with the elective group. Patients in the non-elective group were more often in New York Heart Association (NYHA) class IV on admission, more likely to have concurrent tricuspid regurgitation grade III–IV, more likely to be frail, and were at higher surgical risk. Median levels of high-sensitivity troponin T (45.5 versus 22.3 pg/mL; *p* < 0.001) and N-terminal pro-B-type natriuretic peptide (2233 versus 835 pg/mL; *p* < 0.001) were significantly higher in non-elective patients compared with elective patients.

**Table 1:** Baseline characteristics

	Total (*n* = 512)	Elective (*n* = 378)	Non-elective (*n* = 134)	*P*-value
Age [years]	82.0 (78.7–85.3)	81.8 (78.7–84.9)	83.4 (78.4–86.0)	0.067
Female, n [%]	237 (46.3)	165 (43.7)	72 (53.7)	0.044
BMI [kg/m^2^]	26.1 (23.5–29.4)	26.0 (23.8–29.3)	26.7 (23.4–30.7)	0.399
CAD, n [%]	322 (62.9)	238 (63.0)	84 (62.7)	0.955
COPD, n [%]	59 (11.5)	43 (11.4)	16 (11.9)	0.860
CVD, n [%]	66 (12.9)	43 (11.4)	23 (17.2)	0.086
STS-score [%]	3.1 (2.3–4.9)	3.0 (2.2–4.4)	4.2 (2.5–5.9)	< 0.001
Diabetes mellitus, n [%]	170 (33.2)	117 (31.0)	53 (39.6)	0.069
Dyslipidaemia, n [%]	316 (61.7)	228 (60.3)	88 (65.7)	0.273
History of AF, n [%]	219 (42.8)	151 (39.9)	68 (50.7)	0.030
Hypertension, n [%]	473 (92.4)	347 (91.8)	126 (94.0)	0.403
NYHA class IV on admission	59 (11.5)	30 (7.9)	29 (21.6)	< 0.001
PAD, n [%]	42 (8.2)	30 (7.9)	12 (9.0)	0.712
PH (sPAP > 55 mmHg), n [%]	76 (14.8)	49 (13.0)	27 (20.1)	0.049
LVEF < 55%, n [%]	204 (39.8)	158 (41.8)	46 (34.3)	0.129
Prev. cardiac surgery, n [%]	87 (17.0)	62 (16.4)	25 (18.7)	0.550
Frailty syndrome^a^, n [%]	150 (29.3)	61 (16.1)	89 (66.4)	< 0.001
eGFR < 60 mL/min/1.73m^2^, n [%]	311 (60.7)	210 (55.6)	101 (75.4)	< 0.001
Creatinine [µmol/L]	100 (82–133)	95 (81–124)	115 (93–154)	< 0.001
hs-TNT [pg/mL]	25.6 (15.4–45.1)	22.3 (14.8–37.7)	45.5 (24.7–115.4)	< 0.001
NT-proBNP [pg/mL]	1072 (433–2770)	835 (398–2036)	2233 (924–5539)	< 0.001
AVA [cm^2^]	0.8 (0.6–0.9)	0.8 (0.6–0.9)	0.7 (0.6–0.9)	0.286
MPG [mmHg]	38 (29–48)	38 (29–47)	38 (28–50)	0.901
MR III–IV, n [%]	12 (2.3)	6 (1,6)	6 (4,5)	0.089
TR III–IV, n [%]	19 (3.7)	9 (2.4)	10 (7.5)	0.014

*Legend:* Values are presented as counts (percentages) or median (IQR)

*AF* atrial fibrillation, *AVA* aortic valve area, *BMI* body mass index, *CAD* coronary artery disease, *COPD* chronic obstructive pulmonary disease, *CVD* cerebrovascular disease, *eGFR* estimated glomerular filtration rate, *hs-TNT* high-sensitivity troponin T, *LVEF* left ventricular ejection fraction, *MPG* mean pressure gradient, *MR* mitral regurgitation, *NT-proBNP* N-terminal pro-B-type natriuretic peptide, *NYHA* New York Heart Association, *PAD* peripheral artery disease, *PH* pulmonary hypertension, *Prev.* previous, *sPAP* systolic pulmonary artery pressure, *STS* Society of Thoracic Surgeons, *TR* tricuspid regurgitation

^a^Frailty syndrome was clinically diagnosed without specific frailty assessment tools

### Procedure-related variables and outcomes

**Fig. 1:**
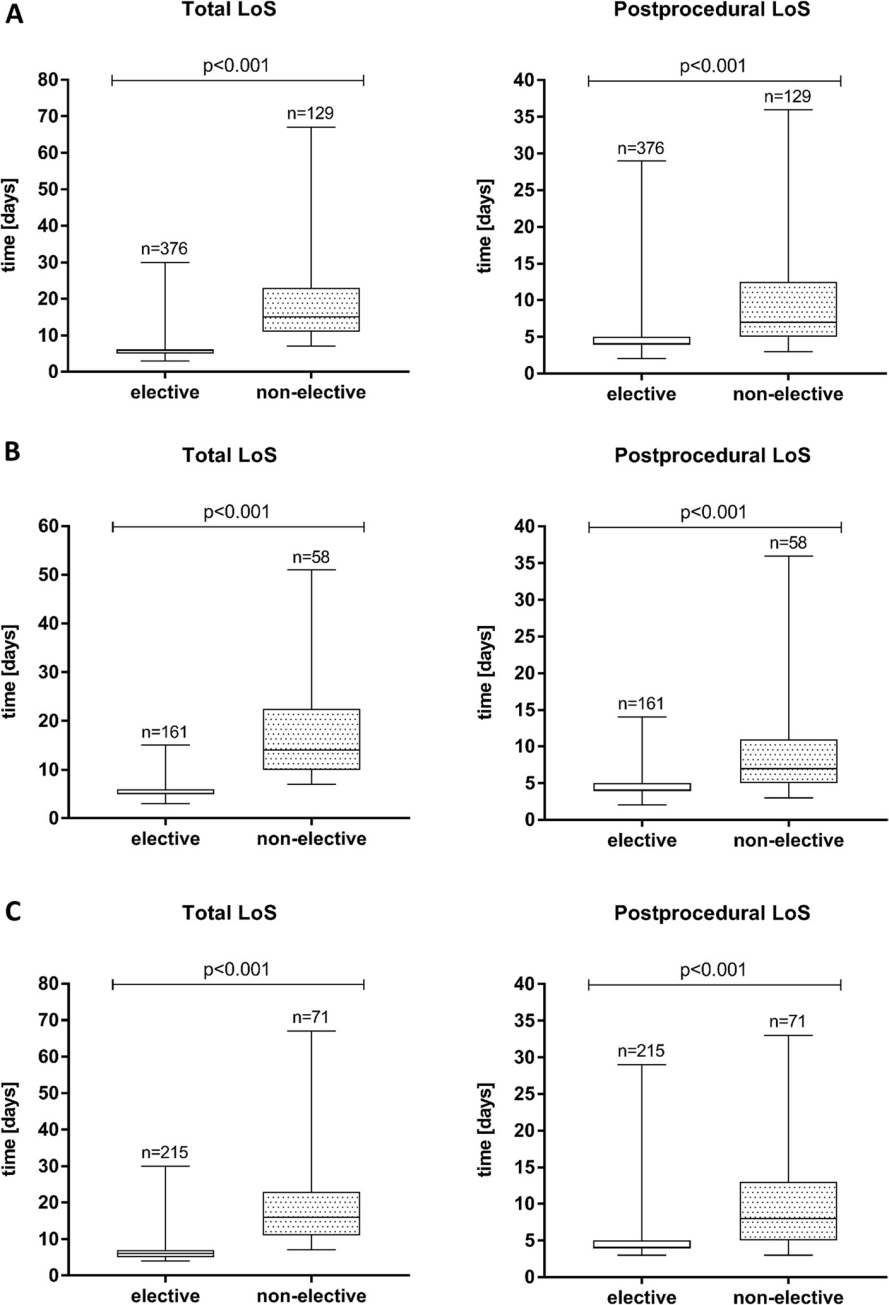
Total length of stay and postprocedural length of stay after TAVI presented as boxplots. **A** Comparison between elective and non-elective patients (all valve types). **B** Comparison between elective and non-elective patients (SAPIEN platform only). **C** Comparison between elective and non-elective patients (CoreValve platform only)

Overall, 43.6% of patients received a balloon-expandable valve and 56.4% received a self-expanding valve using transfemoral (percutaneous) access; the valve type did not differ significantly between the elective and non-elective groups (Table 2). The median duration of the procedure did also not differ between groups. The median duration of hospitalization (from admission to discharge) was 6 days for the total study population, including a median time from procedure to discharge of 5 days. Patients who underwent elective TAVI had a shorter hospital stay compared with the non-elective group (median time from admission to discharge 6 vs. 15 days), including a shorter postprocedural stay (median time from procedure to discharge 4 versus 7 days) (Fig. 1A). This effect was consistent between different heart valves (SAPIEN and CoreValve platform) (Fig. 1B and C). Most patients who underwent an elective procedure were discharged home, whereas almost half of those who underwent a non-elective procedure were discharged to rehabilitation care.

**Table 2:** Procedure-related variables and outcomes

	Total (*n* = 512)	Elective (*n* = 378)	Non-elective (*n* = 134)	*P*-value
Procedural duration [min]	46 (37–56)	46 (37–55)	46 (37–57)	0.414
Procedural contrast agent [mL]	79 (62–99)	78 (64–98)	80 (57–100)	0.727
Balloon-expandable valve, n [%]	223 (43.6)	162 (42.9)	61 (45.5)	0.613
Self-expanding valve, n [%]	289 (56.4)	216 (57.1)	73 (54.5)	0.613
Time: admission to discharge [days]	6 (5–9)	6 (5–6)	15 (11–23)	< 0.001
Time: procedure to discharge [days]	5 (4–6)	4 (4–5)	7 (5–13)	< 0.001
Discharge to home, n [%]	422 (82.4)	356 (94.2)	66 (49.3)	< 0.001
Discharge to rehabilitation, n [%]	81 (15.8)	18 (4.8)	63 (47.0)	< 0.001
**VARC-3**				
New PPI, n [%]	74 (14.5)	47 (12.4)	27 (20.1)	0.029
New LBBB, n [%]	88 (17.2)	75 (19.8)	13 (9.7)	0.008
New RBBB, n [%]	8 (1.6)	7 (1.9)	1 (0.7)	0.375
Stroke with disability, n [%]	4 (0.8)	2 (0.5)	2 (1.5)	0.281
Type 3 (life-threatening) bleeding, n [%]	29 (5.7)	17 (4.5)	12 (9.0)	0.055
Myocardial infarction, n [%]	0 (0)	0 (0)	0 (0)	-
Conversion to open surgery, n [%]	4 (0.8)	2 (0.5)	2 (1.5)	0.281
AKIN stage 3/4, n [%]	2 (0.4)	0 (0)	2 (1.5)	-
Death at 30 days, n [%]	9 (1.8)	4 (1.1)	5 (3.7)	0.030*
MACE at 30 days, n [%]	13 (2.5)	6 (1.6)	7 (5.2)	0.021*
Death at 1 year, n [%]	44 (8.6)	19 (5.0)	25 (18.7)	< 0.001*

*Legend:* Values are presented as counts (percentages) or median (IQR)

*AKIN* Acute Kidney Injury Network, *LBBB* left bundle branch block, *MACE* Major adverse cardiovascular events, *PPI* permanent pacemaker implantation, *RBBB* right bundle branch block, *VARC-3* Valve Academic Research Consortium-3

^*^p-value was calculated using the log-rank test

**Fig. 2:**
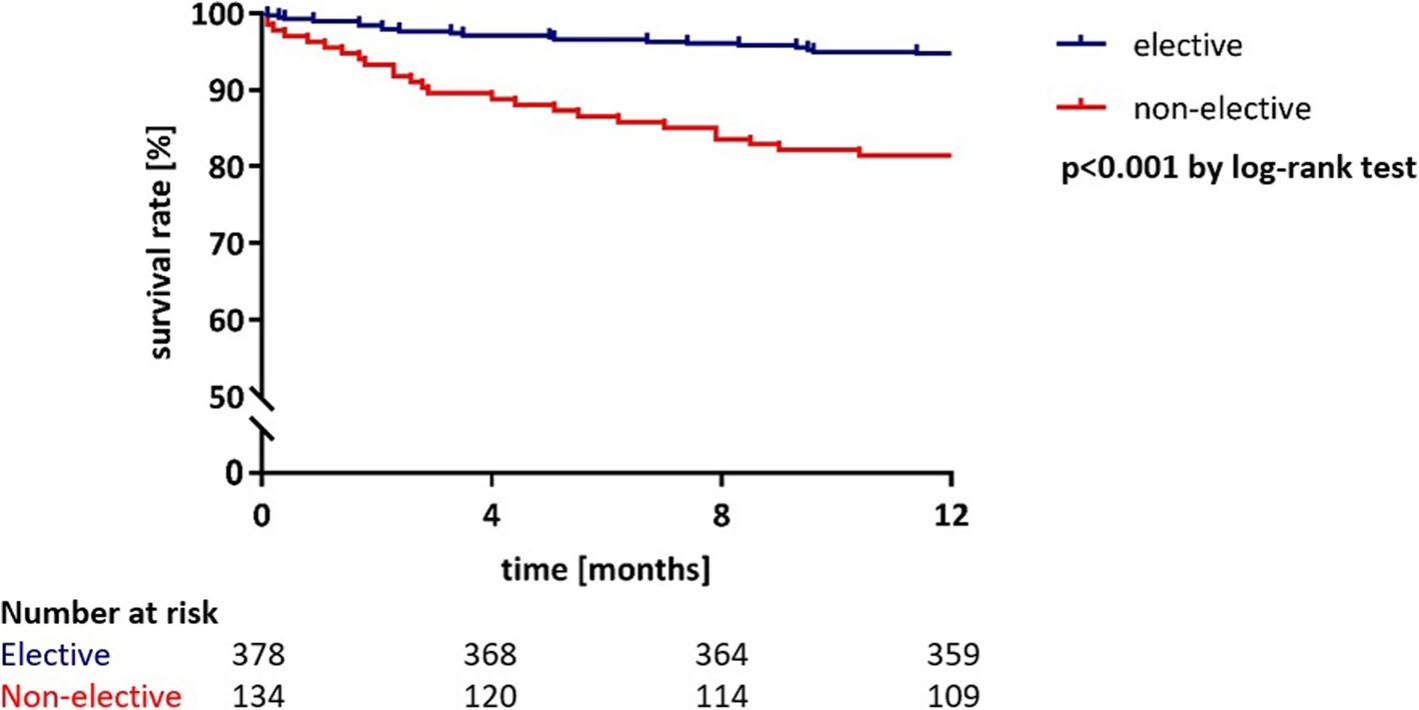
Comparison of the survival rate over 12 months between elective and non-elective patients

In the total study population, the most common VARC-3 complications were new left bundle branch block (LBBB; 17.2%), new permanent pacemaker implantation (PPI; 14.5%) and type 3 (life-threatening) bleeding (5.7%). LBBB was more common in the elective group compared with the non-elective group, whereas new PPI was more often observed in the non-elective group than in the elective group. The overall mortality rate at 30 days post-procedure was 1.8%; the rate was lower in the elective group than in the non-elective group (1.1% versus 3.7%; *p* = 0.03). In addition, major adverse cardiovascular events (MACE) at 30 days, defined as a composite endpoint of cardiovascular death, non-fatal stroke and non-fatal myocardial infarction, were less frequently observed in the elective group (1.6% versus 5.2%, *p* = 0.021) (Table 2). All-cause mortality at 1-year after TAVI occurred in 19 patients (5.0%) in the elective group compared to 25 patients (18.7%) in the non-elective group (*p* < 0.001) (Fig. 2).

### Fast-track TAVI protocol

**Fig. 3:**
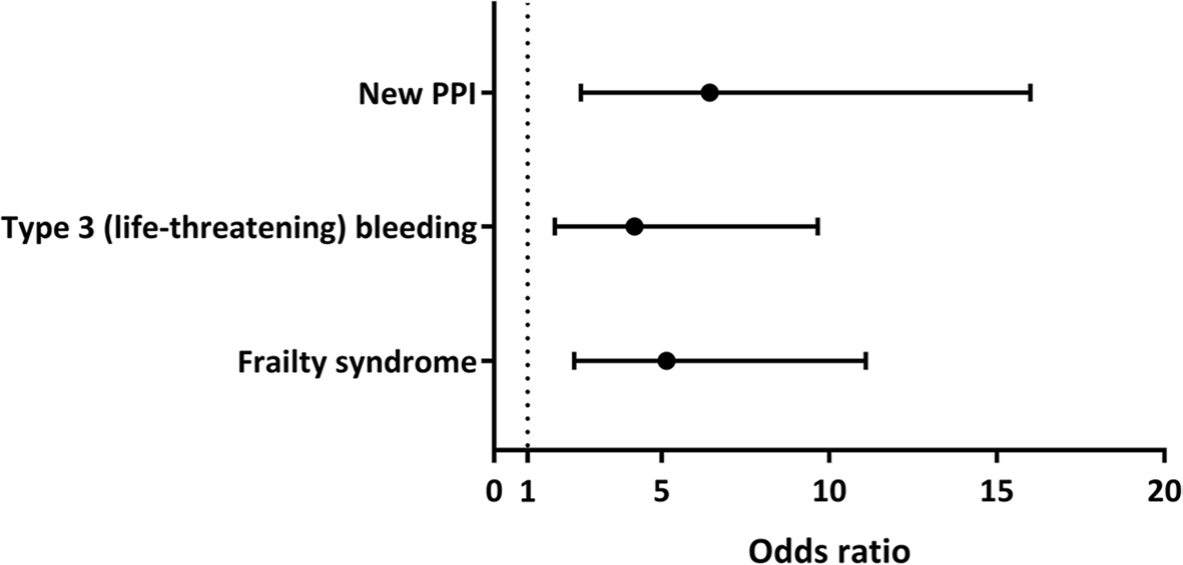
Factors associated with a failure of the TAVI protocol (total length of stay ≤ 5 days)

Crude and adjusted ORs for factors associated with non-adherence to the fast-track TAVI protocol are summarised in Table 3 and Fig. 3**.** The presence of frailty syndrome and renal impairment at baseline were associated with a reduced likelihood of adhering to the fast-track protocol (OR for non-fast-track TAVI 6.20; 95% CI 2.95–13.00; *p* < 0.001, and 1.55; 95% CI 1.03–2.34; *p* = 0.035, respectively). Significant procedural complications with a negative impact on the fast-track protocol included new PPI (OR for non-elective TAVI 6.96; 95% CI 2.88–16.84; *p* < 0.001), new bundle branch block or AF (OR 1.78; 95% CI 1.07–2.94; *p* = 0.026), life-threatening bleeding complications (OR 3.53; 95% CI 1.57–7.93; *p* = 0.002) and the use of self-expanding valves (OR 1.57; 95% CI 1.04–2.38; *p* = 0.031). After multivariate adjustment, the OR for new permanent PPI was 6.44 (95% CI 2.59–16.00), the OR for type 3 (life threatening) bleeding was 4.19 (95% CI 1.82–9.66) and the OR for frailty was 5.15 (95% 2.40–11.09).

**Table 3:** Factors associated with TAVI fast-track protocol failure

	Fast-Track (*n* = 172)	No fast-track (*n* = 204)	Crude OR (95% CI)	*P*-value	Adjusted OR (95% CI)	*p*-value
***Baseline characteristics***						
Frailty syndrome^a^, n [%]	9 (5.2)	52 (25.5)	6.20 (2.95–13.00)	< 0.001	5.15 (2.40–11.09)	< 0.001
eGFR < 60 mL/min/1.73m^2^, n [%]	85 (49.4)	123 (60.3)	1.55 (1.03–2.34)	0.035	0.87 (0.54–1.38)	0.550
***Procedural factors***						
New PPI, n [%]	6 (3.5)	41 (20.1)	6.96 (2.88–16.84)	< 0.001	6.44 (2.59–16.00)	< 0.001
NBBB or atrial fibrillation, n [%]	29 (16.9)	54 (26.5)	1.78 (1.07–2.94)	0.026	1.59 (0.92–2.75)	0.100
Type 3 (life-threatening) bleeding, n [%]	8 (4.7)	30 (14.7)	3.53 (1.57–7.93)	0.002	4.19 (1.82–9.66)	< 0.001
Self-expanding valve, n [%]	88 (51.2)	127 (62.3)	1.57 (1.04–2.38)	0.031	1.35 (0.86–2.13)	0.191

*Legend:* Results are presented as crude and adjusted odds ratios (OR) with 95% confidence interval (CI) for non-fast-track versus fast-track TAVI

*eGFR* estimated glomerular filtration rate, *NBBB* new bundle branch block, *PPI* permanent pacemaker implantation, *TAVI* transcatheter aortic valve implantation

^a^Frailty syndrome was clinically diagnosed without specific frailty assessment tools

## Discussion

The main findings of this study were that: (1) non-elective TAVI patients had acceptable mortality rates at 30 days, but disproportionately worse outcomes at 1 year compared to elective TAVI patients; (2) approximately half of patients (54.5%) scheduled for elective TAVI could not be discharged early due to baseline (frailty syndrome) and periprocedural factors (new PPI and life-threatening bleeding complications).

### Elective versus non-elective patients

Patients undergoing TAVI at our centre had a median age of 82 years. Self-expanding valves (56.4%) were more often used than balloon-expandable valves (43.6%). Approximately three-quarters of patients underwent elective procedures. Patients who underwent elective TAVI had less comorbidities including frailty syndrome and were at lower surgical risk compared to non-elective patients. The median duration of hospitalisation (from admission to discharge) for the total study population was 6 days. Overall, most elective patients were discharged home, whereas nearly half of those who underwent a non-elective procedure were discharged into rehabilitative care. The patient population described in the current study is consistent with further research from Germany, as well as from other European countries [10, 12, 13]. These reports also reflect the increased morbidity of patients in need for non-elective TAVI while there is some disagreement on whether it may have an impact on outcomes [6, 14]. Interestingly, non-elective patients had a longer preprocedural length of stay (median 8 days) compared to the postprocedural length of stay (median 7 days). This clearly relates to the urgency of admission of non-elective patients usually requiring heart failure treatment for acute decompensation and the need for a thorough diagnostic work-up, planning and scheduling of the TAVI procedure. However, this finding also suggests that further improvements in the preprocedural care of non-elective patients as part of a fast-track protocol may significantly reduce the total length of stay in this patient group, e.g. by means of a streamlined preprocedural diagnostic workup and a standardized heart failure treatment protocol. In addition, it seems intuitive that a shorter preprocedural length of stay may also have a positive impact on the postprocedural length of stay, e.g. by reducing the delirium risk.

Previously published data from “The Society of Thoracic Surgeons and the American College of Cardiology Transcatheter Valve Therapy (STS/ACC TVT) Registry” indicated that non-elective TAVI is associated with an increase in periprocedural complication rates as well as 30-day and 1-year mortality compared to elective TAVI [5]. The authors reported a 30-day mortality of 4.3% in elective versus 8.7% in non-elective patients (*p* < 0.001) and a 1-year mortality rate of 17.5% in elective compared to 29.1% in non-elective patients (*p* = 0.001). The study included procedures performed during the years 2011–2016 which significantly limits the applicability of the results to contemporary clinical practice. In addition, patients had a high comorbidity burden and the majority of cases were done in general anaesthesia. A recent study from the United Kingdom also investigated outcomes of elective versus non-elective TAVI patients [6]. The authors found that mortality rates at 30-days (3.5% in elective versus 3.3% in non-elective patients, *p* = 0.81) and after 1 year (10.9% versus 11.0%, *p* = 0.81) were similar between both groups.

Compared to the aforementioned two studies, elective patients in our cohort had low mortality rates both at 30 days (1.1%) and at 1 year (5.0%). Regarding periprocedural complications, we observed a significant higher rate of new permanent pacemaker implantation and left-bundle block in the non-elective group, which may reflect the higher degree of adverse cardiac remodelling in the non-elective subgroup. Other procedural complications did not differ significantly between both groups. Non-elective patients showed acceptable mortality rates at 30 days (3.7%), which significantly increased at 1 year (18.7%). While a direct comparison with the previous studies is certainly limited by different study designs and definitions, our study indicates that (1) elective TAVI patients show superior outcomes compared to non-elective patients and (2) non-elective TAVI patients constitute a high-risk population. Whether or not specific follow-up strategies and optimised medical treatment may improve outcomes in this patient group has to be determined in future trials.

### Elective TAVI using a fast-track protocol

Elective TAVI can reduce the burden on healthcare resources while maintaining clinical efficacy and safety [15–17]. At our centre, we implemented a fast-track protocol aimed at optimizing the TAVI pathway and limiting the total length of stay to ≤ 5 days for elective patients. In the German healthcare system, 4 full days are currently considered the minimum length of stay necessary to safely perform TAVI by enabling approximately 72 h of postprocedural telemetry monitoring. The median total length of stay for elective patients was 5–6 days. In contrast, patients who underwent non-elective TAVI had a total hospital stay of 11–23 days. In principle, these findings suggest that appropriate patients were selected for elective TAVI. This is highly relevant, as postprocedural length of stay is one of the main factors contributing to the increase in periprocedural costs of TAVI [18, 19]. Studies have shown that some patients may be discharged within 24–72 h after TAVI without compromising safety or outcomes [16, 20–22]. For example, the Vancouver 3 M (Multidisciplinary, Multimodality, but Minimalist) Clinical Pathway algorithm has been shown to enable next-day discharge in a selected TAVI patient population while maintaining good safety and efficacy [20]. In our study, elective patients had a postprocedural stay of 4–5 days. In contrast, non-elective patients had a postprocedural stay of 5–13 days. This suggests that elective patients are being selected and treated appropriately, allowing a shorter postprocedural stay in this patient group. Measures to identify waitlisted patients who are at increased risk for AS decompensation and scheduling them for early elective TAVI could potentially reduce the number of patients requiring urgent, non-elective TAVI. In addition, consideration could be given to measures that might reduce the pre- and especially postprocedural length of stay in patients requiring non-elective TAVI, such as optimized treatment of comorbidities, standardized prevention and management of delirium, implementation of a specialized geriatric physiotherapy programme as well as social evaluation and early transfer to geriatric rehabilitation if needed. Nonetheless, there will always be a proportion of patients who, due to their individual health status and circumstances, require a longer length of stay than others, and it is perhaps more helpful to take the approach that ‘*timely’* discharge rather than ‘*early’* discharge should be the main objective [19].

### Factors affecting fast-track TAVI

As a key finding of our study, 54.5% of elective patients were unable to be discharged early and thus did not meet the fast-track criteria of total length of stay ≤ 5 days. Various predictors of early discharge or delayed discharge after TAVI had previously been reported [16, 23–26]. This was investigated in the COORDINATE pilot study [10] where most patients were discharged after 5 days. Length of hospital stay was affected by the patients’ risk profile, clinical pathways and healthcare reimbursement rules, and early discharge was only appropriate for selected patients. Our centre uses a fast-track approach for elective TAVI patients. The analysis of factors affecting the feasibility of a fast-track approach found that the presence of frailty and renal impairment were associated with a reduced likelihood of following a fast-track pathway. Other studies have also identified markers of frailty and renal dysfunction as predictors of a longer hospital stay or delayed discharge [16, 24, 26]. These patients are likely to require an increased preprocedural work-up and greater support after TAVI, which will potentially increase the length of time in hospital. We also found that the occurrence of postoperative complications, including new PPI, new bundle branch block or AF as well as major bleeding, reduced the likelihood of adherence to the fast-track pathway. Procedure-related complications are likely to increase the length of stay after TAVI [27]. Previous studies have also found that AF [24, 26] and the need for new PPI [16, 28] were associated with longer length of stay/delayed discharge after TAVI. Our analysis also found an association between the use of self-expanding valves and a fast-track approach, yet this was not confirmed in the multivariate analysis. The use of balloon-expandable valves has previously been identified as a predictor of next-day discharge [28], while a sub-analysis of the STS/ACC Transcatheter Valve Therapy registry found that use of self-expanding valves was a predictor of delayed discharge (defined as > 72 h) at US centres [27]. The authors suggested that this could relate to specific technical requirements associated with some self-expanding valves, such as a mandated temporary pacemaker or the use of surgical cut-down. However, these technical limitations have now been widely overcome. As demonstrated in a systematic analysis, the use of self-expanding valves is associated with a higher incidence of conduction disturbances after TAVI compared to balloon-expandable valves which is likely to contribute to a prolonged hospital stay [29]. Future prospective trials comparing contemporary transcatheter heart valves are needed to confirm these findings.

### Limitations

Several limitations of the study must be acknowledged. First, our study is limited by its single-centre, non-randomised design. As a consequence, both measured and unmeasured confounding factors may limit the conclusions that can be drawn from our analysis. In addition, potentially clinically relevant differences between both groups may have been under‐ and overestimated. Future research is needed to improve the outcomes of both elective and non-elective patients. Second, five days is the minimum length of stay required for full reimbursement in the German healthcare system which might substantially limit the applicability of the study findings to other countries. Third, secondary endpoints, such as quality of life and physical capacity, were not accounted for. Fourth, frailty syndrome was clinically diagnosed. Geriatric evaluation and frailty screening tools were not routinely applied. Fifth, a significant proportion of patients had low-gradient AS which is frequently encountered in the TAVI population [1]. Additional parameters such as stroke volume index and Agatston score to investigate subtypes of low-gradient AS were not sufficiently available in this study population.

## Conclusions

Non-elective patients undergoing TAVI had acceptable periprocedural outcomes, but significantly worse outcomes at 1 year compared to elective patients and thus constitute a high-risk population. Implementing a TAVI fast-track protocol has the potential to facilitate rapid mobilisation and early discharge. However, a significant number of patients are unable to be discharged early. Improvements in patient assessment, periprocedural care, follow-up strategies and optimized treatment of both elective and non-elective TAVI patients are needed.

## Data Availability

The datasets for this study will not be made publicly available due to data protection reasons but are available from the corresponding author on reasonable request.

## Abbreviations

AF: Atrial fibrillation
AS: Aortic stenosis
CIs: Confidence intervalf
IQR: Interquartile range
LBBB: Left bundle branch block
MACE: Major adverse cardiovascular events
OR: Odds ratio
PPI: Permanent pacemaker implantation
STS: Society of Thoracic Surgeons
TAVI: Transcatheter aortic valve implantation
VARC-3: Valve Academic Research Consortium-3
